# Evolutionary Origins of Drought Tolerance in Spermatophytes

**DOI:** 10.3389/fpls.2021.655924

**Published:** 2021-06-22

**Authors:** Alexander M. C. Bowles, Jordi Paps, Ulrike Bechtold

**Affiliations:** ^1^School of Life Sciences, University of Essex, Colchester, United Kingdom; ^2^School of Biological Sciences, University of Bristol, Bristol, United Kingdom

**Keywords:** drought tolerance, evolution, green plants, phylogenetics, desiccation

## Abstract

It is commonly known that drought stress is a major constraint limiting crop production. Drought stress and associated drought tolerance mechanisms are therefore under intense investigation with the view to future production of drought tolerant crops. With an ever-growing population and variable climate, novel approaches need to be considered to sustainably feed future generations. In this context, definitions of drought tolerance are highly variable, which poses a major challenge for the systematic assessment of this trait across the plant kingdom. Furthermore, drought tolerance is a polygenic trait and understanding the evolution of this complex trait may inform us about patterns of gene gain and loss in relation to diverse drought adaptations. We look at the transition of plants from water to land, and the role of drought tolerance in enabling this transition, before discussing the first drought tolerant plant and common drought responses amongst vascular plants. We reviewed the distribution of a combined “drought tolerance” trait in very broad terms to encompass different experimental systems and definitions used in the current literature and assigned a binary trait “tolerance vs. sensitivity” in 178 extant plant species. By simplifying drought responses of plants into this “binary” trait we were able to explore the evolution of drought tolerance across the wider plant kingdom, compared to previous studies. We show how this binary “drought tolerance/sensitivity” trait has evolved and discuss how incorporating this information into an evolutionary genomics framework could provide insights into the molecular mechanisms underlying extreme drought adaptations.

## Introduction

### Defining Drought Tolerance

Water is essential for life on Earth, and there are diverse adaptations to water availability within the plant tree of life. This diversity has acted as a barrier to understanding broad evolutionary patterns of drought and desiccation tolerance. Vegetative desiccation tolerance was crucial for the colonization of terrestrial habitats, where desiccation tolerance is defined as the ability of plants to survive for extended periods of time in the absence of suspending metabolism, preventing oxidative damage and maintaining the native structures of macromolecules and membranes under extreme water limitations (Oliver et al., 2020).

On the other hand, adaptations to water deficits in species that are unable to survive periods of desiccation (desiccation sensitive species) encompass very different ecological strategies including drought escape, avoidance or tolerance (Kooyers, 2015; Basu et al., 2016). Drought escape is indicated by rapid growth and early flowering to reproduce before the onset of terminal drought. Drought avoidance limits growth during periods of dehydration by lowering stomatal conductance, transpiration, and maintenance of high tissue water content (Levitt, 1980; Kooyers, 2015), and drought tolerance is the ability to endure low tissue water through maintenance of cell turgor by osmotic adjustment (Morgan, 1984). Drought resistance, on the other hand, facilitates plant survival during periods of dehydration, but does not contribute toward growth and yield maintenance post drought stress (Blum, 2005, 2009; Passioura, 2007). Interestingly, it is the drought avoidance strategies designed to evade damage or death, which have been proposed to drive the evolution of genes controlling growth in stressful environments (Maggio et al., 2018). This means that plant mechanisms to cope with stressful environments during evolution were mostly designed to avoid detrimental effects such as injury and death, and consequently extinction.

Furthermore, drought tolerance is a highly diverse and complex trait with confusion arising over interchangeable terms used in the literature including drought tolerance, -resistance, -avoidance or –escape. This makes defining a “drought tolerant” plant a fairly subjective exercise (Bechtold, 2018). Drought tolerance is most commonly used by plant scientists to collectively describe plants that are able to grow after a period of low water availability (Maggio et al., 2018). However, when drought stress is induced in a lab/agricultural setting, timescales can range from hours/days to weeks, or months and include many different induction methods (Passioura, 2007). As such investigating the broad scale evolution of drought tolerance traits is motivated by the need to understand drought tolerance strategies for different types of drought conditions, which can vary in induction method, severity, and timing (Monroe et al., 2018).

### Water Availability as a Driver of Plant Evolution

The relationships of the plant kingdom (Viridiplantae) with water have changed dramatically. The common ancestor of extant green plants were photosynthetic eukaryotes adapted to life in aquatic environments (Delwiche and Cooper, 2015). Subsequently green plants diverged into Chlorophyta (green algae) and Streptophyta (land plants and streptophyte algae), approximately one billion years ago (Morris et al., 2018). Streptophyte evolutionary innovations enabled responses to novel environmental challenges including extremes of UV, temperature, and light (de Vries and Archibald, 2018; De Vries et al., 2018). Streptophyte algae are found in a range of brackish, freshwater, and terrestrial habitats, which demonstrates their diversity of adaptations to water availability and other stressors such as desiccation, salinity, pH and nutrient variation (Delwiche and Cooper, 2015; de Vries et al., 2016; Fürst-Jansen et al., 2020). It has been remarked that the adaptations needed for plants on land and shallow or transient water are highly similar (Becker et al., 2020; Donoghue and Paps, 2020).

Studies of streptophyte algae reveal that features once thought to be unique to land plants, in fact first appeared earlier in the ancestor of close algal relatives (e.g., associations with substrate microbiota) (de Vries and Archibald, 2018; De Vries et al., 2018; Nishiyama et al., 2018; Cheng et al., 2019; Liang et al., 2019; Wang et al., 2019). To a certain extent, the ancestors of Streptophyta (and subgroups e.g., Phragmoplastophyta) displayed traits that would facilitate later the transition to life on land (Delaux et al., 2015). For example, analysis of streptophyte genomes has revealed that ancestral charophytes acquired the fundamental machinery for land plant adaptation including hormone signaling, high light, and desiccation tolerance (Hori et al., 2014; Nishiyama et al., 2018; Cheng et al., 2019; Wang et al., 2019). Consequently, the common genes in streptophyte algae and land plants represent the ancestral gene pool from which embryophyte genes with functions in desiccation and drought responses have evolved.

Based on the latest fossil evidence and molecular dating, the first plants transitioned from aquatic to terrestrial environments approximately 500 million years ago (mya) in the Ordovician—Cambrian period (Rubinstein et al., 2010; Morris et al., 2018). All extant land plants descend from a single common ancestor (Wickett et al., 2014; de Vries and Archibald, 2018) and have since diversified into almost 400,000 species that have shaped modern ecosystems (Kenrick and Crane, 1997; Willis, 2017). Their rise to ecological dominance has enabled plants to colonize every continent on Earth which involves adaptations to extreme environments including arid deserts (Xiao et al., 2015; Copetti et al., 2017) and the Antarctic (Lee et al., 2014). The origin of the first embryophytes was accompanied by the production of novel developmental and morphological mechanisms for adaptation to life on land (e.g., the alternation between haploid and diploid generations, three dimensional growth, cuticle development; Bowman et al., 2017). Analysis of fossils from the Rhynie Chert, a well maintained fossil deposit in Scotland, suggests that in the Early Devonian (∼400 mya) plants were tolerant to high salt levels and osmotic stress, a key component of drought stress (Channing and Edwards, 2009).

Whole genome sequencing of species on either side of the transition to land is revealing much about the genetic innovations accompanying the development of desiccation tolerance in land plants (Bowman et al., 2017; De Clerck et al., 2018; de Vries and Archibald, 2018; De Vries et al., 2018; Nishiyama et al., 2018; Cheng et al., 2019; Wang et al., 2019; Jiao et al., 2020; Li et al., 2020; Zhang et al., 2020). For example, it is now evident that the backbone of phytohormone signaling, required for stress responses, either predates or accompanies the transition to land (Wang et al., 2015, 2019; Bowman et al., 2017; Bowles et al., 2020; Cannell et al., 2020). This means that although many key genes evolved prior to the transition to land, specific responses and genetic re-wiring of stress response pathways occurred later in land plant evolution, allowing for greater adaptive plasticity to water availability.

It has been shown that the responses of extant bryophytes have changed very little to those of early land plants (Oliver et al., 2005). For example, desiccation tolerance in bryophytes is common with over 200 of 2,100 bryophyte species capable of this phenotype (Proctor et al., 2007; Wood, 2007b; Gao et al., 2017). Therefore, desiccation tolerance is believed to be an ancestral trait in embryophytes and a key component for the adaptations for life on land (Oliver et al., 2000; Wood, 2007a). Importantly these plants would have lacked the ability to regulate water content, termed poikilohydry (Stevenson et al., 2016; Becker et al., 2020). In tracheophytes, or vascular plants, desiccation tolerance is less common. The responses to limited water availability in early vascular plants diversified by increasing regulatory and morphological complexity (Lu et al., 2020), and their origin was accompanied by the appearance of a sporophyte dominant life cycle and vascular tissue (Harrison, 2017). These two innovations enabled plants to tolerate dry conditions and to control the internal movement of water and nutrients. This suggests that during the evolution of tracheophytes, early forms of drought tolerance originated. In the lycophytes, the majority of species are susceptible to desiccation, although a few tolerant species have been identified including *Selaginella lepidophylla* (Yobi et al., 2013) and *Selaginella tamariscina* (Wang et al., 2010). In flowering plants, only 160 of 369,000 species have been confirmed as desiccation tolerant (Wood, 2007b; Royal Botanic Gardens Kew, 2016). Based on evolutionary thinking, this implies that desiccation tolerance was lost in the ancestor of tracheophytes, being replaced by drought tolerance.

### Reconstructing the Evolutionary History of Drought Tolerance Across the Plant Kingdom

Many important physiological, structural, and regulatory responses to drought have arisen during the evolutionary history of plants. For example, morphological innovations linked to drought tolerance include stomata, roots, vascular tissue, specialized reproduction, waxy cuticle, euphylls, and seeds (Harrison, 2017). To the best of our knowledge, the broad scale evolutionary history of drought tolerance has never been investigated, with work completed only at the species or genus level (Iseki et al., 2018). Analyzing the distribution of drought and desiccation tolerance in plants could therefore be useful for our understanding of the origins of drought tolerance traits as well as the evolution of land plants. To make a comment about the evolution of drought tolerance across the plant phylogeny, a simplified definition of a “drought tolerant” plant is developed.

Due to the interest in modern plant genomics, plant species with genomic representation were curated to understand the evolution of drought tolerance. As genomic data is being produced at increasingly high rates, not all genomes were included in our analysis. As such, we used the selection of species for which good quality genomes are available as detailed in Bowles et al. (2020). Therefore, this study aims to determine a combined “drought tolerance” trait encompassing definitions commonly used in the literature for 178 plant species. This was mapped onto a species tree to reconstruct the evolutionary history of the combined “drought tolerance” trait. With this information about the broad scale evolutionary patterns of this combined “drought tolerance” trait, we outline the future directions for improving our understanding of the molecular mechanisms of extreme drought tolerance.

It has recently been shown that salt tolerance has independently evolved in land plants having important implications for evolutionary biology and the breeding of stress-tolerant crops (Flowers et al., 2010). In contrast, evidence from fossils and molecular inferences about phytohormone evolution suggest that there was a common adaptation to drought in the ancestor of land plants (Channing and Edwards, 2009; Cheng et al., 2019; Sun et al., 2019; Bowles et al., 2020). Additionally, the land plant ancestor acquired many exaptations to terrestrial stresses experienced during the transition from water to land (de Vries and Archibald, 2018; De Vries et al., 2018; Fürst-Jansen et al., 2020). Therefore, it is hypothesized that a single origin of the combined “drought tolerance” trait will be inferred in the ancestor of land plants or earlier.

## Materials and Methods

### Defining Drought Tolerance and Drought Sensitivity

Using a literature search, we assign a putative drought tolerant/sensitive status to each species based on a literature search in relation to each species’ name (Supplementary Table 1 and Supplementary Data 1). The combined drought tolerance trait contained also included desiccation tolerance (Supplementary Table 1). Short of experimentally characterizing species individually, as completed for species in the genus Vigna (Iseki et al., 2016, 2018), this is an approach that can provide a simplified binary definition where a plant is either drought tolerant or drought sensitive for a range of well characterized but also less studied plants. Additionally, a domestication status was designated by querying the genome paper of each plant genome in the study for wild (wild) and domesticated (cultivated, cultured, domesticated) terms.

### Concatenation Approach to Build a Calibrated Tree

To complete the ancestral state reconstruction to infer the evolution of drought tolerance, a species tree with branch lengths was required. A fixed topology was produced based on the NCBI taxonomy database (Federhen, 2012), which is consistent with recent publications on plant phylogenetics (Leebens-Mack et al., 2019). To infer the branch lengths of this tree, genes from 171 Homology Groups (HGs) present in all Archaeplastida were extracted from the computational pipeline described in Bowles et al., 2020. Briefly, HGs are groups of proteins clustered by graph theory approaches and assumed to share a most recent common ancestor. Specifically, we selected HGs present in all Archaeplastida species, which ensures there is no missing data either for genes or for taxa (Supplementary Data 2, 3). Due to the broad clustering of homology groups (Bowles et al., 2020), each HG contained more than one protein sequence per species. For gene tree inference, we selected the first gene for each species for each HG. We selected the concatenation approach, incorporating phylogenetic inference from multiple gene alignments, to provide a strong phylogenetic signal with which to build a robust species tree (Wickett et al., 2014). Selected genes from each HG were individually aligned using MAFFT with –auto parameter (Katoh et al., 2002) and trimmed using trimal using the -automated1 option (Capella-Gutiérrez et al., 2009). PhyUtility was used to concatenate all genes into a supermatrix (Smith and Dunn, 2008). Once a concatenated supermatrix was produced, a partition file was created which was used to identify each gene alignment (Supplementary Data 4). A species tree was inferred using IQTree, with parameters altered to determine different rates of sequence evolution for each individual gene alignment and the constraint tree outlined above used as a guide tree (Nguyen et al., 2015). The final species tree produced was used in subsequent analysis.

### Ancestral State Reconstruction

**FIGURE 1:**
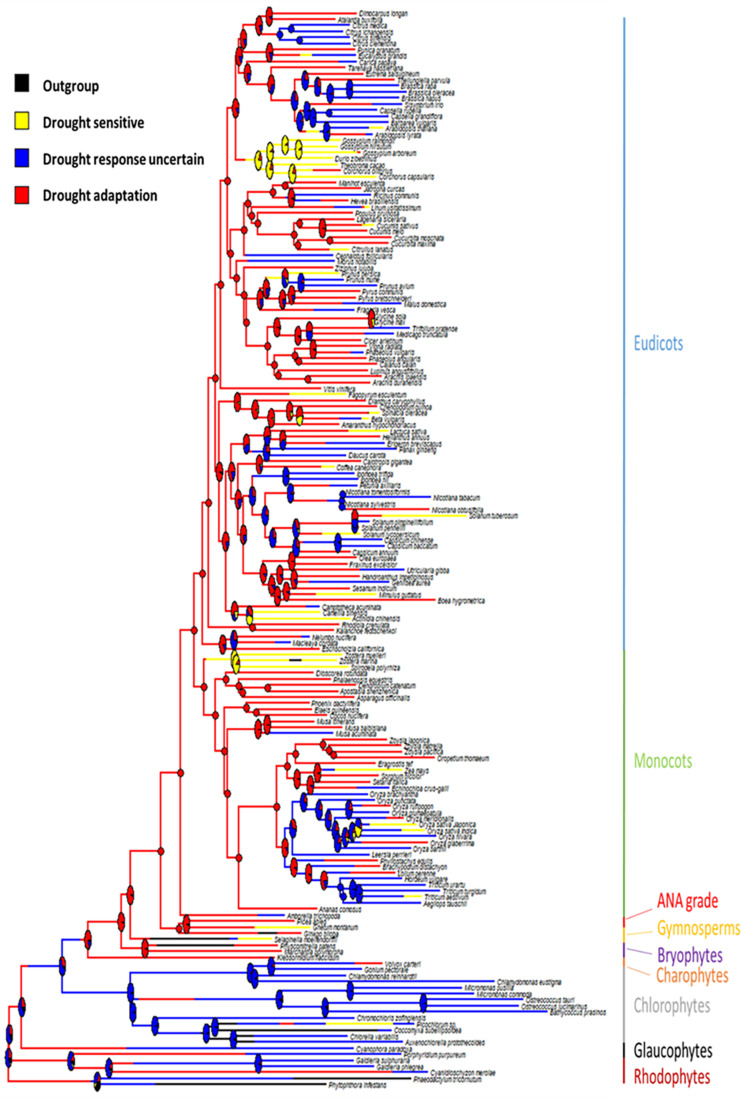
Ancestral state reconstruction of drought adaptation on a species tree. Branches are colored by drought response status. Pie charts represent the support for the ancestral state at each internal node.

**FIGURE 2:**
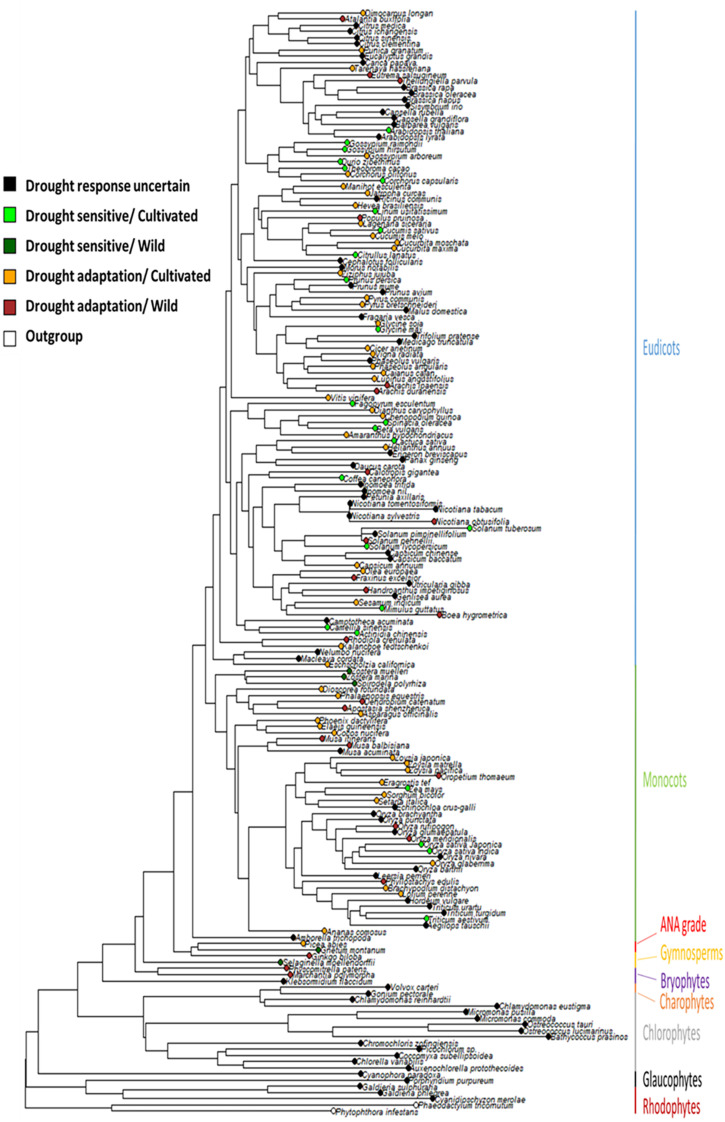
Distribution of drought adaptation and domestication on a species tree. Terminal nodes indicate the status of each species drought adaptation combined with their domestication status.

Phytools were used to estimate ancestral character states for discretely valued traits (e.g., drought tolerance) using a continuous-time Markov chain model (Revell, 2012). The MCMC approach is used to sample character histories from their posterior probability distribution, termed stochastic character mapping (Huelsenbeck et al., 2003). To sample a greater portion of the distribution of the character history, 100 stochastic maps were produced and summarized (Figure 1). Additionally, a collective character, incorporating domestication status combined with the “drought tolerance” trait, was mapped onto the same tree (Figure 2).

## Results

### Ancestral State Reconstruction of Drought Tolerance

Of the 178 plant species included in this analysis, 74 were recognized as drought tolerant, 29 were identified as drought sensitive and for 75 no clear definition could be assigned. These species are distributed across the plant phylogeny, occurring in all major evolutionary groups, although there is a bias on the number of genomes available toward angiosperms. Ancestral state reconstruction of the collective “drought tolerance” trait was completed for all green plants using the calibrated tree (Figure 1). The analysis suggested that the last common ancestor (LCA) of Streptophyta was drought adapted and likely had the capacity for desiccation tolerance.

Furthermore, our analysis also revealed that the LCA of vascular plants was likely drought adapted (Figure 1). This suggests that an adaptation to drought tolerance was acquired once, potentially with the development of vascular tissue and a sporophyte dominated lifestyle. Importantly, drought tolerance was subsequently highly retained, which suggests that for any drought sensitive species that appear later than this ancestor, the ability to adapt to drought has been lost. For example, *Spirodela polyrhiza* (duckweed) (Wang et al., 2014), *Zostera marina* (Olsen et al., 2016) and *Zostera muelleri* (Lee et al., 2016) are in the order Alismatales and have all adapted to an aquatic lifestyle (i.e., *Zostera* are a genus of seagrasses). Drought tolerance has been lost in these plants due to their transition back into aquatic environments.

### Distribution of Domesticated and Drought Adapted Species Across the Plant Phylogeny

During domestication, selection can lead to the loss of genetic diversity (Doebley et al., 2006), and indirect effects of domestication may have led to the loss of particular stress tolerance traits including drought tolerance (Yu et al., 2008; Zhu et al., 2019; Wang et al., 2020b,a). In the same manner as drought adaptations above, domestication statuses for the same sets of plant species were assigned which were exclusively sourced from the genome paper of each plant genome. To investigate the impact of domestication on the loss of drought tolerance, the domestication statuses and drought statuses across the species tree were plotted (Figure 2). Drought sensitivity appears to be common amongst many of the major crop species. In fact, drought sensitivity is predominantly found in crop species, suggesting domestication could explain the loss of drought tolerance. The only cases of loss of drought tolerance in wild species were in Alismatales and in non-flowering plants, *Selaginella moellendorffii* and *Gnetum montanum*. The ancestral states of the LCA of land plants were drought tolerant and wild (Figure 2). Therefore, for any plants that are drought tolerant and cultivated, this represents the ancestral state for their drought adaptation.

## Discussion

Due to the variability of the “drought tolerance” definition, it is challenging to carry out a systematic assessment of drought tolerance to not only provide insights into trait distribution but also the evolution across the plant kingdom. Evidence of drought-tolerance mostly comes from observations under natural growing conditions (biogeography) of a species, or from controlled and field-based experiments (literature). The validity of this empirical data relies on careful phenotyping over the life cycle of the various plant species, and the number and type of variables used, all of which can confound a single definition of drought tolerance. There is little data available that compares the rigor with which drought tolerance has been assessed and defined across independent studies and species, and therein lies the problem. The definition of drought tolerance is not only highly ambiguous (Bechtold, 2018; Maggio et al., 2018; Tardieu et al., 2018) it is also sparse across the plant phylogeny.

In this work, a combined “drought tolerance” trait was developed for such a broad taxonomic range of plant species by querying the literature in reference to each species in the genomic dataset (Bowles et al., 2020). This combined “drought tolerance” trait, therefore, produces a simplified binary outcome, where a plant is either drought tolerant or drought sensitive. In simplifying the states of drought tolerance, it enabled us to investigate the broad scale evolutionary patterns across the plant phylogeny, identifying the origin of this common trait in the ancestor of vascular plants. However, by simplifying the trait and utilizing such a broad range of species, this approach has led to a large proportion of “unclassified” plant species, which clearly added uncertainty to the ancestral state reconstruction in the early nodes. Once in land plants almost all the transitions observed are from drought tolerance to sensitivity where many drought sensitive species are surrounded by drought tolerant species (Figure 1). This suggests that at least the emergence of drought sensitivity is not affected by the number of unclassified plant species, but it also suggests that drought-sensitivity may be a more reliable and easy to define trait compared to drought tolerance, which emerged prior to land plants and was subsequently lost in few lineages (Figure 1).

Alternative approaches to defining drought tolerance for a broad range of taxa are slowly beginning to emerge. For example, the 2020 release of the TRY database, a global database of curated plant traits, investigated the prevalence of species tolerance to drought which incorporated a low, medium, and high level of tolerance for a broader range of taxa (Kattge et al., 2020), but does not cover all the species in our genomic dataset (Bowles et al., 2020). Currently, a major constraint with this database is that there are three arbitrary categories of drought tolerance (low, medium, and high), which are as yet not clearly defined as a phenotypic trait. An additional approach for defining a collective “drought tolerance” trait could be to investigate the geographical distribution of drought tolerance. Some genome papers provide information about the geographical location of the plant material used to sequence the plant genome. For some species, this data is listed as longitude and latitude coordinates, for example, plant material for the *Zostera marina* genome was sourced from Fårö Island, Sweden (latitude: 59° 55.234′ N, longitude: 21° 47.766′ E, Olsen et al., 2016). Additionally, the global occurrence and severity of drought has been investigated (Sheffield and Wood, 2008) and is continuously monitored (Hao et al., 2014). With information about the occurrence of geographical historical and current drought events plant species could be classified based on their location in drought prone regions. However, there are limitations with this approach, for example, plant material sampled from a botanic garden or grown in a laboratory outside a plant’s natural geographical range.

In producing the broad definition for a diverse range of taxa presented here, this leads to a reduction of the complexity of drought tolerance, which has its limitations. By producing this simplified binary definition, the trait omits the intricacies and nuances of drought adaptations. Additionally, drought adaptations, in wild populations, demonstrate a scale of responses, which are not captured by the definition presented here. Even within species, drought responses can vary, as shown by analysis of European Arabidopsis populations (Exposito-Alonso et al., 2017). In spite of these caveats, this work is the most phylogenetically comprehensive study to date to investigate the evolution of drought tolerance.

## Conclusion and Future Directions

Despite the limitations given above, we demonstrate that drought adapted plants are present across the plant phylogeny, highlighting how plant relationships with water have changed over the last 700 million years. The LCA of vascular plants was likely drought tolerant. Despite a baseline level of drought adaptation, extreme responses to drought have arisen during plant evolution. For example, the capacity of *Boea hygrometrica* as a resurrection plant (Xiao et al., 2015) and the adaptation of the desert tree *Populus pruinosa* (Yang et al., 2017).

Importantly, our analyses highlighted the distribution of drought sensitivity in many important crop species, which appears common for stress tolerance traits with evolutionary loss following the domestication of crop plants (Mayrose et al., 2011; Koziol et al., 2012). This suggests that “undomesticated” plant species are a useful source to identify “novel,” or rediscover “old” genes for improving stress tolerance. Indeed, crop wild relatives are considered to be a pool of genetic resources for engineering stress tolerant crops. Consequently, understanding the evolutionary emergence and loss of drought tolerance will provide an important basis for breeding the crops of the future. For example, evolutionary studies, which reconstruct the origin and development of drought tolerance in a variety of plant lineages, may help us to understand why plant breeding has failed to produce a range of productive drought tolerant crops.

With this in mind, examining the distribution of drought responses across the plant phylogeny may shed light on shared genes and functions. By exploring the genetic framework underlying these traits in the context of plant evolution, the genes and changes in sequences responsible for diverse adaptations can be illuminated. By incorporating stress tolerance traits, evolutionary biology and plant genomes, stress tolerant gene identification based on evolutionary genomic analysis may be possible, and may also help us develop new drought-tolerant lines by revealing the order of gene gains and losses, or indicating genetic backgrounds in which drought tolerance may be developed for the future. This work therefore sits in the backdrop of the pressures of sustainably feeding a growing global population and the negative impacts of climate change. Therefore, novel approaches such as those presented here, are required to feed future generations.

This approach may be even more powerful in light of the vast array of genomic data becoming available for a wide range of plant species. Further taxonomic revision of our datasets including species that have been released since the original study in 2020 (Bowles et al., 2020) will help toward our understanding of how drought tolerance/sensitivity has evolved across the plant kingdom, but only if they can be classified as tolerant or sensitive. To make real progress, common reporting standards and databases which allow for the interoperability of phenotypic traits need to be developed (Ćwiek-Kupczyńska et al., 2016; Ely et al., 2021), not just for drought tolerance traits but also associated metadata, linked to clear trait definitions. Descriptions of experimental details such as timing, growth stages, induction method and domestication status as well as the huge variety of phenotypic parameters that are used to classify and define drought stress responses need to be integrated in a way that standardizes trait definitions across the different plant science disciplines.

Clear definitions linked to drought tolerance/sensitivity would therefore enable a better reconstruction of ancestral states and comparison across species and environmental contexts. Therefore, the reconciliation of already existing genome and phenotype data requires further investigation and collaborative community efforts, in order to facilitate the application of large-scale evolutionary genomics projects.

## Data Availability Statement

The original contributions presented in the study are included in the article/Supplementary Material, further inquiries can be directed to the corresponding author/s.

## Author Contributions

AB performed the analysis. AB, JP, and UB wrote the manuscript. All authors listed have made a substantial, direct and intellectual contribution to the work, and approved it for publication.

## Conflict of Interest

The authors declare that the research was conducted in the absence of any commercial or financial relationships that could be construed as a potential conflict of interest.

## Supplementary Material

Supplementary Table 1 Terms used to identify drought responses in plants.
