# Supplementary material for: Evolutionary Origins of Drought Tolerance in Spermatophytes
Source: Front Plant Sci. 2021 Jun 22;12:655924. doi: 10.3389/fpls.2021.655924 (PMC8258419; doi:10.3389/fpls.2021.655924)
Supplement: Supplementary Table 1 — Terms used to identify drought responses in plants. [file Data_Sheet_1.zip › Supplementary Table 1.DOCX]

**Supplementary Table 1**. Terms used to identify drought responses in plants

| Drought adapted | Drought sensitive |
| --- | --- |
| Drought tolerance | Drought sensitive |
| Drought avoidance | Drought susceptible |
| Drought escape | Drought prone |
| Desiccation tolerance |  |
