# Supplementary material for: Evolutionary Origins of Drought Tolerance in Spermatophytes
Source: Front Plant Sci. 2021 Jun 22;12:655924. doi: 10.3389/fpls.2021.655924 (PMC8258419; doi:10.3389/fpls.2021.655924)
Supplement: Supplementary Table 1 — Terms used to identify drought responses in plants. [file Data_Sheet_1.zip › Supplementary_Data_1_Bowles et al.docx]

A list of the Archaeplastida species in the genomic dataset, a four-letter species code, a drought adaptation status, the literature for any drought status and the cultivation status. For the drought adaptation status, plants were characterised as drought adapted, drought sensitive or for plants, with no clear response drought response uncertain. For the cultivation status, plant were defined as wild, cultured or cultivated. These were defined based on evidence from the genome papers of these species.

| **Species** | **Species Code** | **Drought status** | **Source** | **Cultivation** |
| --- | --- | --- | --- | --- |
| *Galdieria sulphuraria* | gsul | Drought response uncertain | NA | Cultivated |
| *Galdieria phlegrea* | gphl | Drought response uncertain | NA | Wild |
| *Cyanidioschyzon merolae* | cmer | Drought response uncertain | NA | Cultured |
| *Porphyridium purpureum* | ppur | Drought response uncertain | NA | Cultured |
| *Cyanophora paradoxa* | cpar | Drought response uncertain | NA | Cultured |
| *Bathycoccus prasinos* | bpra | Drought response uncertain | NA | Wild |
| *Ostreococcus lucimarinus* | oluc | Drought response uncertain | NA | Cultured |
| *Ostreococcus tauri* | otau | Drought response uncertain | NA | Cultured |
| *Micromonas commoda* | mcom | Drought response uncertain | NA | Cultured |
| *Micromonas pusilla* | mpus | Drought response uncertain | NA | Cultured |
| *Auxenochlorella protothecoides* | apro | Drought response uncertain | NA | Cultured |
| *Chlorella variabilis* | cvar | Drought response uncertain | NA | Cultured |
| *Picochlorum sp.* | psp. | Drought response uncertain | NA | Cultivated |
| *Coccomyxa subellipsoidea* | csub | Drought response uncertain | NA | Cultured |
| *Chromochloris zofingiensis* | czof | Drought response uncertain | NA | Cultured |
| *Gonium pectorale* | gpec | Drought response uncertain | NA | Cultured |
| *Volvox carteri* | vcar | Drought adaptation | (Jaenicke and Gilles, 1982) | Cultured |
| *Chlamydomonas reinhardtii* | crei | Drought response uncertain | NA | Cultured |
| *Chlamydomonas eustigma* | ceus | Drought response uncertain | NA | Wild |
| *Klebsormidium flaccidum* | kfla | Drought adaptation | (Holzinger and Becker, 2015) | Cultured |
| *Marchantia polymorpha* | mpol | Drought adaptation | (Bowman et al., 2017) | Wild |
| *Physcomitrella patens* | ppat | Drought adaptation | (Li et al., 2017b) | Wild |
| *Selaginella moellendorffii* | some | Drought sensitive | (Dinakar and Bartels, 2013) | Wild |
| *Ginkgo biloba* | gbil | Drought adaptation | (Shan-An et al., 1997) | Wild |
| *Picea abies* | pabi | Drought adaptation | (Kohler et al., 2010) | Cultivated |
| *Gnetum montanum* | gmon | Drought sensitive | (Wan et al., 2018a) | Wild |
| *Amborella trichopoda* | atri | Drought response uncertain | NA | Wild |
| *Spirodela polyrhiza* | spol | Drought sensitive | (Cheng, 2011) | Wild |
| *Zostera marina* | zmar | Drought sensitive | (Leuschner and Ellenberg, 2017) | Wild |
| *Zostera muelleri* | zmue | Drought sensitive | (Lee et al., 2016) | Wild |
| *Dioscorea rotundata* | drot | Drought adaptation | (Loko et al., 2015) | Cultivated |
| *Apostasia shenzhenica* | ashe | Drought adaptation | (Zhang et al., 2017) | Wild |
| *Dendrobium catenatum* | dcat | Drought adaptation | (Wan et al., 2018b) | Wild |
| *Phalaenopsis equestris* | pequ | Drought adaptation | (Wan et al., 2018b) | Cultivated |
| *Asparagus officinalis* | aoff | Drought adaptation | (Whitmore, 2000) | Cultivated |
| *Phoenix dactylifera* | pdac | Drought adaptation | (Safronov et al., 2017) | Cultivated |
| *Elaeis guineensis* | egui | Drought adaptation | (Murugesan et al., 2017) | Cultivated |
| *Cocos nucifera* | cnus | Drought adaptation | (Gomes and Prado, 2007) | Cultivated |
| *Ananas comosus* | acom | Drought adaptation | (Ming et al., 2015) | Cultivated |
| *Oryza brachyantha* | obra | Drought response uncertain | NA | Wild |
| *Oryza punctata* | opun | Drought response uncertain | NA | Wild |
| *Oryza glumipatula* | oglu | Drought response uncertain | NA | Wild |
| *Oryza rufipogon* | oruf | Drought adaptation | (Biaolin et al., 2010) | Wild |
| *Oryza meridionalis* | omer | Drought adaptation | (Vaughan et al., 2003) | Wild |
| *Oryza barthii* | obar | Drought response uncertain | NA | Wild |
| *Oryza glaberrima* | ogla | Drought adaptation | (Bimpong et al., 2011) | Cultivated |
| *Oryza nivara* | oniv | Drought response uncertain | NA | Wild |
| *Oryza sativa Indica* | osai | Drought sensitive | (Wei et al., 2016) | Cultivated |
| *Oryza sativa Japonica* | osaj | Drought sensitive | (Wei et al., 2016) | Cultivated |
| *Leersia perrieri* | lper | Drought response uncertain | NA | Wild |
| *Phyllostachys edulis* | pedu | Drought adaptation | (Wu et al., 2018b) | Wild |
| *Brachypodium distachyon* | bdis | Drought adaptation | (Bertolini et al., 2013) | Cultivated |
| *Hordeum vulgare* | hvul | Drought response uncertain | NA | Cultivated |
| *Aegilops tauschii* | atau | Drought response uncertain | NA | Cultivated |
| *Triticum urartu* | tura | Drought response uncertain | NA | Cultivated |
| *Triticum aestivum* | taes | Drought sensitive | (He et al., 2009) | Cultivated |
| *Triticum turgidum* | ttur | Drought response uncertain | NA | Wild |
| *Lolium perenne* | lpee | Drought adaptation | (Cheplick et al., 2000) | Cultivated |
| *Echinochloa crus-galli* | ecru | Drought response uncertain | NA | Wild |
| *Setaria italica* | sita | Drought adaptation | (Li et al., 2014) | Cultivated |
| *Zea mays* | zmay | Drought sensitive | (Agrama and Moussa, 1996) | Cultivated |
| *Sorghum bicolor* | sbic | Drought adaptation | (Abdel-Ghany et al., 2020) | Cultivated |
| *Eragrostis tef* | etef | Drought adaptation | (Degu et al., 2008) | Cultivated |
| *Zoysia japonica* | zjap | Drought adaptation | (Patton et al., 2017) | Cultivated |
| *Zoysia matrella* | zmat | Drought adaptation | (Ntoulas et al., 2012) | Cultivated |
| *Zoysia pacifica* | zpac | Drought adaptation | (Patton et al., 2017) | Cultivated |
| *Oropetium thomaeum* | otho | Drought adaptation | (Vanburen et al., 2015) | Wild |
| *Musa itinerans* | miti | Drought adaptation | (Wu et al., 2018a; Kew Science, 2020b) | Wild |
| *Musa balbisiana* | mbal | Drought adaptation | (Nansamba et al., 2020) | Wild |
| *Musa acuminata* | macu | Drought response uncertain | NA | Wild |
| *Eschscholzia californica* | ecal | Drought adaptation | (Wilts et al., 2018) | Cultivated |
| *Macleaya cordata* | mcor | Drought response uncertain | NA | Wild |
| *Nelumbo nucifera* | nnuc | Drought response uncertain | NA | Cultivated |
| *Kalanchoe fedtschenkoi* | kfed | Drought adaptation | (Yang et al., 2017b) | Cultivated |
| *Rhodiola crenulata* | rcre | Drought adaptation | (Zhang et al., 2019) | Wild |
| *Vitis vinifera* | vvin | Drought adaptation | (Gambetta et al., 2020) | Cultivated |
| *Lupinus angustifolius* | lang | Drought adaptation | (Jensen and Henson, 1990; Kalandyk et al., 2017) | Cultivated |
| *Arachis duranensis* | adur | Drought adaptation | (Guimarães et al., 2012) | Wild |
| *Arachis ipaensis* | aipa | Drought adaptation | (Azevedo Neto et al., 2010) | Wild |
| *Cajanus cajan* | ccaj | Drought adaptation | (Varshney et al., 2012) | Cultivated |
| *Phaseolus angularis* | pang | Drought adaptation | (Cortés et al., 2013) | Cultivated |
| *Phaseolus vulgaris* | pvul | Drought response uncertain | NA | Cultivated |
| *Vigna radiata* | vrad | Drought adaptation | (Iseki et al., 2018) | Cultivated |
| *Glycine max* | gmax | Drought sensitive | (Wang et al., 2017) | Cultivated |
| *Glycine soja* | gsoj | Drought adaptation | (Ji et al., 2010) | Cultivated |
| *Cicer arietinum* | cari | Drought adaptation | (Varshney et al., 2014) | Cultivated |
| *Medicago truncatula* | mtru | Drought response uncertain | NA | Cultivated |
| *Trifolium pratense* | tpra | Drought response uncertain | NA | Cultivated |
| *Fragaria vesca* | fves | Drought response uncertain | NA | Cultivated |
| *Prunus avium* | pavi | Drought response uncertain | NA | Cultivated |
| *Prunus mume* | pmum | Drought response uncertain | NA | Cultivated |
| *Prunus persica* | pper | Drought sensitive | (Eldem et al., 2012) | Cultivated |
| *Pyrus bretschneideri* | pbre | Drought adaptation | (Cao et al., 2018) | Cultivated |
| *Pyrus communis* | pcom | Drought adaptation | (Paudel et al., 2019) | Cultivated |
| *Malus domestica* | mdom | Drought response uncertain | NA | Cultivated |
| *Ziziphus jujuba* | zjuj | Drought adaptation | (Cruz et al., 2012) | Cultivated |
| *Morus notabilis* | mnot | Drought response uncertain | NA | Wild |
| *Cucurbita maxima* | cmax | Drought adaptation | (Yasar et al., 2014) | Cultivated |
| *Cucurbita moschata* | cmos | Drought adaptation | (Cao et al., 2017) | Cultivated |
| *Citrullus lanatus* | clan | Drought sensitive | (Zhang et al., 2011) | Cultivated |
| *Lagenaria siceraria* | lsic | Drought adaptation | (Mashilo et al., 2017) | Cultivated |
| *Cucumis melo* | cmel | Drought adaptation | (Kusvuran, 2012) | Cultivated |
| *Cucumis sativus* | csat | Drought sensitive | (Wang et al., 2012) | Cultivated |
| *Populus trichocarpa* | ptri | Drought adaptation | (Tang et al., 2015) | Cultivated |
| *Populus pruinosa* | ppru | Drought adaptation | (Yang et al., 2017a) | Wild |
| *Linum usitatissimum* | lusi | Drought sensitive | (Dash et al., 2014) | Cultivated |
| *Jatropha curcas* | jcur | Drought adaptation | (Sapeta et al., 2016) | Cultivated |
| *Manihot esculenta* | mesc | Drought adaptation | (Okogbenin et al., 2013) | Cultivated |
| *Hevea brasiliensis* | hbra | Drought adaptation | (Kew Science, 2020a; Plants for a Future, 2020) | Cultivated |
| *Ricinus communis* | rcom | Drought response uncertain | NA | Cultivated |
| *Cephalotus follicularis* | cfol | Drought response uncertain | NA | Wild |
| *Punica granatum* | pgra | Drought adaptation | (Catola et al., 2016) | Cultivated |
| *Eucalyptus grandis* | egra | Drought response uncertain | NA | Wild |
| *Dimocarpus longan* | dlon | Drought adaptation | (Wiriya-Alongkorn et al., 2013) | Cultivated |
| *Atalantia buxifolia* | abux | Drought adaptation | (Newton and Goodin, 1989) | Wild |
| *Citrus clementina* | ccle | Drought response uncertain | NA | Cultivated |
| *Citrus sinensis* | csin | Drought response uncertain | NA | Cultivated |
| *Citrus ichangensis* | cich | Drought response uncertain | NA | Wild |
| *Citrus medica* | cmed | Drought response uncertain | NA | Cultivated |
| *Carica papaya* | cpap | Drought response uncertain | NA | Cultivated |
| *Tarenaya hassleriana* | thas | Drought adaptation | (Kocacinar, 2015) | Cultivated |
| *Eutrema salsugineum* | esal | Drought adaptation | (Yang et al., 2013) | Wild |
| *Thellungiella parvula* | tpar | Drought adaptation | (Griffith et al., 2007) | Wild |
| *Brassica napus* | bnap | Drought response uncertain | NA | Cultivated |
| *Brassica oleracea* | bole | Drought response uncertain | NA | Cultivated |
| *Brassica rapa* | brap | Drought response uncertain | NA | Cultivated |
| *Sisymbrium irio* | siri | Drought response uncertain | NA | Wild |
| *Barbarea vulgaris* | bvug | Drought response uncertain | NA | Wild |
| *Capsella rubella* | crub | Drought response uncertain | NA | Cultivated |
| *Capsella grandiflora* | cgra | Drought response uncertain | NA | Wild |
| *Arabidopsis thaliana* | atha | Drought sensitive | (Marín‐de la Rosa et al., 2019) | Cultivated |
| *Arabidopsis lyrata* | alyr | Drought response uncertain | NA | Wild |
| *Theobroma cacao* | tcac | Drought sensitive | (Bae et al., 2008) | Cultivated |
| *Corchorus capsularis* | ccap | Drought sensitive | (Yang et al., 2017c) | Cultivated |
| *Corchorus olitorius* | coli | Drought adaptation | (Yang et al., 2017c) | Cultivated |
| *Durio zibethinus* | dzib | Drought sensitive | (Wan Nazri et al., 2014) | Cultivated |
| *Gossypium arboreum* | garb | Drought adaptation | (Maqbool et al., 2009) | Cultivated |
| *Gossypium hirsutum* | ghir | Drought sensitive | (Li et al., 2017a) | Cultivated |
| *Gossypium raimondii* | grai | Drought sensitive | (Chen et al., 2013) | Cultivated |
| *Fagopyrum esculentum* | fesc | Drought sensitive | (Jamwal et al., 2015) | Cultivated |
| *Dianthus caryophyllus* | dcay | Drought adaptation | (Wan et al., 2015) | Cultivated |
| *Beta vulgaris* | bvul | Drought sensitive | (Pidgeon et al., 2006) | Cultivated |
| *Spinacia oleracea* | sole | Drought sensitive | (Schwab and Heber, 1984) | Cultivated |
| *Chenopodium quinoa* | cqui | Drought adaptation | (Al-Naggar et al., 2017) | Cultivated |
| *Amaranthus hypochondriacus* | ahyp | Drought adaptation | (Sunil et al., 2014) | Cultivated |
| *Camptotheca acuminata* | cacu | Drought response uncertain | NA | Wild |
| *Camellia sinensis* | csie | Drought sensitive | (Liu et al., 2016) | Cultivated |
| *Actinidia chinensis* | achi | Drought sensitive | (Mills et al., 2009) | Cultivated |
| *Lactuca sativa* | lsat | Drought sensitive | (Kizil et al., 2012) | Cultivated |
| *Erigeron breviscapus* | ebre | Drought response uncertain | NA | Cultivated |
| *Helianthus annuus* | hann | Drought adaptation | (Badouin et al., 2017) | Cultivated |
| *Panax ginseng* | pgin | Drought response uncertain | NA | Cultivated |
| *Daucus carota* | dcar | Drought response uncertain | NA | Cultivated |
| *Coffea canephora* | ccan | Drought sensitive | (Cheserek and Gichimu, 2012) | Cultivated |
| *Calotropis gigantea* | cgig | Drought adaptation | (Tezara et al., 2011; Mutwakil et al., 2017) | Wild |
| *Fraxinus excelsior* | fexc | Drought adaptation | (Dobrowolska et al., 2011) | Wild |
| *Olea europaea* | oeur | Drought adaptation | (Sofo, 2011) | Cultivated |
| *Boea hygrometrica* | bhyg | Drought adaptation | (Xiao et al., 2015) | Wild |
| *Mimulus guttatus* | mgut | Drought sensitive | (Hughes et al., 2001) | Cultivated |
| *Sesamum indicum* | sind | Drought adaptation | (Golestani and Pakniyat, 2015; Dossa et al., 2017) | Cultivated |
| *Handroanthus impetiginosus* | himp | Drought adaptation | (Dombroski et al., 2014) | Wild |
| *Genlisea aurea* | gaur | Drought response uncertain | NA | Wild |
| *Utricularia gibba* | ugib | Drought response uncertain | NA | Wild |
| *Ipomoea nil* | inil | Drought response uncertain | NA | Cultivated |
| *Ipomoea trifida* | itri | Drought response uncertain | NA | Wild |
| *Petunia inflata* | pinf | Drought response uncertain | NA | Cultivated |
| *Petunia axillaris* | paxi | Drought response uncertain | NA | Cultivated |
| *Nicotiana obtusifolia* | nobt | Drought adaptation | (Su et al., 2017) | Wild |
| *Nicotiana sylvestris* | nsyl | Drought response uncertain | NA | Cultivated |
| *Nicotiana tabacum* | ntab | Drought response uncertain | NA | Cultivated |
| *Nicotiana tomentosiformis* | ntom | Drought response uncertain | NA | Wild |
| *Solanum lycopersicum* | slyc | Drought sensitive | (Mishra et al., 2016) | Cultivated |
| *Solanum pennellii* | spen | Drought adaptation | (Egea et al., 2018) | Wild |
| *Solanum pimpinellifolium* | spim | Drought response uncertain | NA | Wild |
| *Solanum tuberosum* | stub | Drought sensitive | (Boguszewska-Mańkowska et al., 2018) | Cultivated |
| *Capsicum annuum* | cann | Drought adaptation | (Sahitya et al., 2019) | Cultivated |
| *Capiscum baccatum* | cbac | Drought response uncertain | NA | Cultivated |
| *Capiscum chinense* | cchi | Drought response uncertain | NA | Cultivated |

**References**

Abdel-Ghany, S. E., Ullah, F., Ben-Hur, A., and Reddy, A. S. N. (2020). Transcriptome analysis of drought-resistant and drought-sensitive sorghum (Sorghum bicolor) genotypes in response to peg-induced drought stress. *Int. J. Mol. Sci.* 21. doi:10.3390/ijms21030772.

Agrama, H. A. S., and Moussa, M. E. (1996). Mapping QTLs in breeding for drought tolerance in maize (Zea mays L.). *Euphytica* 91, 89–97. doi:10.1007/BF00035278.

Al-Naggar, A., El-Salam, R., Badran, A., and El-Moghazi, M. (2017). Drought tolerance of Five Quinoa (Chenopodium quinoa Willd.) Genotypes and Its Association with Other Traits under Moderate and Severe Drought Stress. *Asian J. Adv. Agric. Res.* 3, 1–13. doi:10.9734/ajaar/2017/37216.

Azevedo Neto, A. D., Nogueira, R. J. M. C., Melo Filho, P. A., and Santos, R. C. (2010). Physiological and biochemical responses of peanut genotypes to water deficit. *J. Plant Interact.* 5, 1–10. doi:10.1080/17429140902999243.

Badouin, H., Gouzy, J., Grassa, C. J., Murat, F., Staton, S. E., Cottret, L., et al. (2017). The sunflower genome provides insights into oil metabolism, flowering and Asterid evolution. *Nature* 546, 148–152. doi:10.1038/nature22380.

Bae, H., Kim, S. H., Kim, M. S., Sicher, R. C., Lary, D., Strem, M. D., et al. (2008). The drought response of Theobroma cacao (cacao) and the regulation of genes involved in polyamine biosynthesis by drought and other stresses. *Plant Physiol. Biochem.* 46, 174–188. doi:10.1016/j.plaphy.2007.10.014.

Bertolini, E., Verelst, W., Horner, D. S., Gianfranceschi, L., Piccolo, V., Inzé, D., et al. (2013). Addressing the role of micrornas in reprogramming leaf growth during drought stress in brachypodium distachyon. *Mol. Plant* 6, 423–443. doi:10.1093/mp/sss160.

Biaolin, H., Shouwu, Y., Yong, W., Zheng, Z., Bingyu, Q., and Jiankun, X. (2010). Drought-resistance identification of dongxiang common wild rice Oryza rufipogon Griff. in whole growth period. *Acta Agron. Sin.* 33, 425–432.

Bimpong, I. K., Serraj, R., Chin, J. H., Ramos, J., Mendoza, E. M. T., Hernandez, J. E., et al. (2011). Identification of QTLs for Drought-Related Traits in Alien Introgression Lines Derived from Crosses of Rice (Oryza sativa cv. IR64) × O. glaberrima under Lowland Moisture Stress. *J. Plant Biol.* 54, 237–250. doi:10.1007/s12374-011-9161-z.

Boguszewska-Mańkowska, D., Pieczyński, M., Wyrzykowska, A., Kalaji, H. M., Sieczko, L., Szweykowska-Kulińska, Z., et al. (2018). Divergent strategies displayed by potato ( *Solanum tuberosum* L.) cultivars to cope with soil drought. *J. Agron. Crop Sci.* 204, 13–30. doi:10.1111/jac.12245.

Bowman, J. L., Kohchi, T., Yamato, K. T., Jenkins, J., Shu, S., Ishizaki, K., et al. (2017). Insights into Land Plant Evolution Garnered from the Marchantia polymorpha Genome. *Cell* 171, 287–304. doi:10.1016/j.cell.2017.09.030.

Cao, H., Wang, L., Nawaz, M. A., Niu, M., Sun, J., Xie, J., et al. (2017). Ectopic Expression of Pumpkin NAC Transcription Factor CmNAC1 Improves Multiple Abiotic Stress Tolerance in Arabidopsis. *Front. Plant Sci.* 8. doi:10.3389/fpls.2017.02052.

Cao, Y., Meng, D., Chen, Y., Abdullah, M., Jin, Q., Lin, Y., et al. (2018). Comparative and Expression Analysis of Ubiquitin Conjugating Domain-Containing Genes in Two Pyrus Species. *Cells* 7. doi:10.3390/cells7070077.

Catola, S., Marino, G., Emiliani, G., Huseynova, T., Musayev, M., Akparov, Z., et al. (2016). Physiological and metabolomic analysis of Punica granatum (L.) under drought stress. *Planta* 243, 441–449. doi:10.1007/s00425-015-2414-1.

Chen, Y., Liu, Z. H., Feng, L., Zheng, Y., Li, D. Di, and Li, X. B. (2013). Genome-wide functional analysis of cotton (Gossypium hirsutum) in response to drought. *PLoS One* 8. doi:10.1371/journal.pone.0080879.

Cheng, T. (2011). NaCl-induced responses in giant duckweed (Spirodela polyrhiza). *J. Aquat. Plant Manag.* 49, 62–71.

Cheplick, G. P., Perera, A., and Koulouris, K. (2000). Effect of drought on the growth of Lolium perenne genotypes with and without fungal endophytes. *Funct. Ecol.* 14, 657–667. doi:10.1046/j.1365-2435.2000.00466.x.

Cheserek, J. J., and Gichimu, B. M. (2012). Drought and heat tolerance in coffee: a review. *Int. Res. J. Agric. Sci. Soil Sci.* 2, 498–501.

Cortés, A. J., Monserrate, F. A., Ramírez-Villegas, J., Madriñán, S., and Blair, M. W. (2013). Drought Tolerance in Wild Plant Populations: The Case of Common Beans (Phaseolus vulgaris L.). *PLoS One* 8. doi:10.1371/journal.pone.0062898.

Cruz, Z. N., Rodríguez, P., Galindo, A., Torrecillas, E., Ondoño, S., Mellisho, C. D., et al. (2012). Leaf mechanisms for drought resistance in Zizyphus jujuba trees. *Plant Sci.* 197, 77–83. doi:10.1016/j.plantsci.2012.09.006.

Dash, P. K., Cao, Y., Jailani, A. K., Gupta, P., Venglat, P., Xiang, D., et al. (2014). Genome-wide analysis of drought induced gene expression changes in flax (Linum usitatissimum). *GM Crops Food* 5, 106–119. doi:10.4161/gmcr.29742.

Degu, H. D., Ohta, M., and Fujimura, T. (2008). Drought tolerance of Eragrostis tef and development of roots. *Int. J. Plant Sci.* 169, 768–775. doi:10.1086/588064.

Dinakar, C., and Bartels, D. (2013). Desiccation tolerance in resurrection plants: New insights from transcriptome, proteome, and metabolome analysis. *Front. Plant Sci.* 4. doi:10.3389/fpls.2013.00482.

Dobrowolska, D., Hein, S., Oosterbaan, A., Wagner, S., Clark, J., and Skovsgaard, J. P. (2011). A review of European ash (Fraxinus excelsior L.): implications for silviculture. *Forestry* 84, 133–148. doi:10.1093/forestry/cpr001.

Dombroski, J. L. D., Freitas, R. M. O. de, Tomczak, V. E., Pinto, J. R. de S., and Farias, R. M. de (2014). Ecophysiology of water stressed Handroanthus impetiginosus (Mart. Ex. DC) Mattos) Seedlings. *Sci. For. Sci.* 42, 155–163.

Dossa, K., Li, D., Wang, L., Zheng, X., Yu, J., Wei, X., et al. (2017). Dynamic transcriptome landscape of sesame (Sesamum indicum L.) under progressive drought and after rewatering. *Genomics Data* 11, 122–124. doi:10.1016/j.gdata.2017.01.003.

Egea, I., Albaladejo, I., Meco, V., Morales, B., Sevilla, A., Bolarin, M. C., et al. (2018). The drought-tolerant Solanum pennellii regulates leaf water loss and induces genes involved in amino acid and ethylene/jasmonate metabolism under dehydration. *Sci. Rep.* 8. doi:10.1038/s41598-018-21187-2.

Eldem, V., Çelikkol Akçay, U., Ozhuner, E., Bakır, Y., Uranbey, S., and Unver, T. (2012). Genome-Wide Identification of miRNAs Responsive to Drought in Peach (Prunus persica) by High-Throughput Deep Sequencing. *PLoS One* 7. doi:10.1371/journal.pone.0050298.

Gambetta, G. A., Herrera, J. C., Dayer, S., Feng, Q., Hochberg, U., and Castellarin, S. D. (2020). The physiology of drought stress in grapevine: Towards an integrative definition of drought tolerance. *J. Exp. Bot.* 71, 4658–4676. doi:10.1093/jxb/eraa245.

Golestani, M., and Pakniyat, H. (2015). Evaluation of Traits Related to Drought Stress in Sesame (Sesamum Indicum L.) Genotypes. *J. Asian Sci. Res.* 5, 465–472. doi:10.18488/journal.2/2015.5.9/2.9.465.472.

Gomes, F. P., and Prado, C. H. B. A. (2007). Ecophysiology of coconut palm under water stress. *Brazilian J. Plant Physiol.* 19, 377–391. doi:10.1590/S1677-04202007000400008.

Griffith, M., Timonin, M., Wong, A. C. E., Gray, G. R., Akhter, S. R., Saldanha, M., et al. (2007). Thellungiella: an Arabidopsis-related model plant adapted to cold temperatures. *Plant. Cell Environ.* 30, 529–538. doi:10.1111/j.1365-3040.2007.01653.x.

Guimarães, P. M., Brasileiro, A. C. M., Morgante, C. V., Martins, A. C. Q., Pappas, G., Silva, O. B., et al. (2012). Global transcriptome analysis of two wild relatives of peanut under drought and fungi infection. *BMC Genomics* 13. doi:10.1186/1471-2164-13-387.

He, L., Gao, Z., and Li, R. (2009). Pretreatment of seed with H 2 O 2 enhances drought tolerance of wheat (Triticum aestivum L.) seedlings. *African J. Biotechnol.* 8, 6151–6157.

Holzinger, A., and Becker, B. (2015). Desiccation tolerance in the streptophyte green alga Klebsormidium: The role of phytohormones. *Commun. Integr. Biol.* 8, 1–4. doi:10.1080/19420889.2015.1059978.

Hughes, R., Bachmann, K., Smirnoff, N., and Macnair, M. R. (2001). The role of drought tolerance in serpentine tolerance in the Mimulus guttatus Fischer ex DC. complex. *S. Afr. J. Sci.* 97, 581–586.

Iseki, K., Takahashi, Y., Muto, C., Naito, K., and Tomooka, N. (2018). Diversity of Drought Tolerance in the Genus Vigna. *Front. Plant Sci.* 9. doi:10.3389/fpls.2018.00729.

Jaenicke, L., and Gilles, R. (1982). “Differentiation and Embryogenesis in Volvox carteri,” in *Biochemistry of Differentiation and Morphogenesis* (Springer Berlin Heidelberg), 288–294. doi:10.1007/978-3-642-68833-1_26.

Jamwal, A., Puri, S., Sharma, S., Bhattacharya, S., and Dhindsa, N. (2015). Polyethylene Glycol Induced Morphological Changes in Fagopyrum esculentum Moench of Indian Himalayan Region. *Asian J. Adv. Basci Sci.* 3, 142–146.

Jensen, C. R., and Henson, I. E. (1990). Leaf Water Relations Characteristics of Lupinus angustifolius and L. cosentinii. *Oecologia* 82, 114–121.

Ji, W., Zhu, Y., Li, Y., Yang, L., Zhao, X., Cai, H., et al. (2010). Over-expression of a glutathione S-transferase gene, GsGST, from wild soybean (Glycine soja) enhances drought and salt tolerance in transgenic tobacco. *Biotechnol. Lett.* 32, 1173–1179. doi:10.1007/s10529-010-0269-x.

Kalandyk, A., Waligórski, P., and Dubert, F. (2017). Role of the maternal effect phenomena in improving water stress tolerance in narrow-leafed lupine (Lupinus angustifolius). *Plant Breed.* 136, 167–173. doi:10.1111/pbr.12457.

Kew Science (2020a). Hevea brasiliensis (Willd. ex A.Juss.) Müll.Arg. *Plants of the World Online*. Available at: http://powo.science.kew.org/taxon/urn:lsid:ipni.org:names:349913-1#descriptions [Accessed December 8, 2020].

Kew Science (2020b). Musa itinerans Cheesman. *Plants of the World Online*. Available at: http://powo.science.kew.org/taxon/urn:lsid:ipni.org:names:584951-1 [Accessed December 8, 2020].

Kizil, Ü., Genc, L., İnalpulat, M., Şapolyo, D., and Mirik, M. (2012). Lettuce (Lactuca sativa L.) yield prediction under water stress using artificial neural network (ANN) model and vegetation indices. *Zemdirbyste* 99, 409–418.

Kocacinar, F. (2015). Photosynthetic, hydraulic and biomass properties in closely related C _3_ and C _4_ species. *Physiol. Plant.* 153, 454–466. doi:10.1111/ppl.12240.

Kohler, M., Sohn, J., Nägele, G., and Bauhus, J. (2010). Can drought tolerance of Norway spruce (Picea abies (L.) Karst.) be increased through thinning? *Eur. J. For. Res.* 129, 1109–1118. doi:10.1007/s10342-010-0397-9.

Kusvuran, S. (2012). Effects of drought and salt stresses on growth, stomatal conductance, leaf water and osmotic potentials of melon genotypes (Cucumis melo L.). *African J. Agric. Res.* 7.

Lee, H. T., Golicz, A. A., Bayer, P. E., Jiao, Y., Tang, H., Paterson, A. H., et al. (2016). The Genome of a Southern Hemisphere Seagrass Species (Zostera muelleri). *Plant Physiol.* 172, 272–283. doi:10.1104/pp.16.00868.

Leuschner, C., and Ellenberg, H. (2017). “Ecology of Central European non-forest vegetation: Coastal to alpine, natural to man-made habitats,” in *Ecology of Central European Non-Forest Vegetation: Coastal to Alpine, Natural to Man-Made Habitats* (Springer International Publishing), 1–1093. doi:10.1007/978-3-319-43048-5.

Li, C., Yue, J., Wu, X., Xu, C., and Yu, J. (2014). An ABA-responsive DRE-binding protein gene from Setaria italica, SiARDP, the target gene of SiAREB, plays a critical role under drought stress. *J. Exp. Bot.* 65, 5415–5427. doi:10.1093/jxb/eru302.

Li, F., Li, M., Wang, P., Cox, K. L., Duan, L., Dever, J. K., et al. (2017a). Regulation of cotton (Gossypium hirsutum) drought responses by mitogen-activated protein (MAP) kinase cascade-mediated phosphorylation of GhWRKY59. *New Phytol.* 215, 1462–1475. doi:10.1111/nph.14680.

Li, Q., Zhang, X., Lv, Q., Zhu, D., Qiu, T., Xu, Y., et al. (2017b). Physcomitrella patens dehydrins (PpDHNA and PpDHNC) confer salinity and drought tolerance to transgenic arabidopsis plants. *Front. Plant Sci.* 8. doi:10.3389/fpls.2017.01316.

Liu, S. C., Jin, J. Q., Ma, J. Q., Yao, M. Z., Ma, C. L., Li, C. F., et al. (2016). Transcriptomic analysis of tea plant responding to drought stress and recovery. *PLoS One* 11. doi:10.1371/journal.pone.0147306.

Loko, Y. L., Adjatin, A., Dansi, A., Vodouhè, R., and Sanni, A. (2015). Participatory evaluation of Guinea yam (Dioscorea cayenensis Lam.–D. rotundata Poir. complex) landraces from Benin and agro-morphological characterization of cultivars tolerant to drought, high soil moisture and chips storage insects. *Genet. Resour. Crop Evol.* 62, 1181–1192. doi:10.1007/s10722-015-0221-y.

Maqbool, A., Abbas, W., Rao, A. Q., Irfan, M., Zahur, M., Bakhsh, A., et al. (2009). Gossypium arboreum GHSP26 enhances drought tolerance in Gossypium hirsutum. *Biotechnol. Prog.* 26. doi:10.1002/btpr.306.

Marín‐de la Rosa, N., Lin, C., Kang, Y. J., Dhondt, S., Gonzalez, N., Inzé, D., et al. (2019). Drought resistance is mediated by divergent strategies in closely related Brassicaceae. *New Phytol.* 223, 783–797. doi:10.1111/nph.15841.

Mashilo, J., Odindo, A. O., Shimelis, H. A., Musenge, P., Tesfay, S. Z., and Magwaza, L. S. (2017). Drought tolerance of selected bottle gourd [Lagenaria siceraria (Molina) Standl.] landraces assessed by leaf gas exchange and photosynthetic efficiency. *Plant Physiol. Biochem.* 120, 75–87. doi:10.1016/j.plaphy.2017.09.022.

Mills, T. M., Li, J., and Behboudian, M. H. (2009). Physiological Responses of Gold Kiwifruit (Actinidia chinensis) to Reduced Irrigation. *J. Am. Soc. Hortic. Sci.* 134, 677–683.

Ming, R., VanBuren, R., Wai, C. M., Tang, H., Schatz, M. C., Bowers, J. E., et al. (2015). The pineapple genome and the evolution of CAM photosynthesis. *Nat. Genet.* 47, 1435–1442. doi:10.1038/ng.3435.

Mishra, U., Rai, A., Kumar, R., Singh, M., and Pandey, H. P. (2016). Gene expression analysis of Solanum lycopersicum and Solanum habrochaites under drought conditions. *Genomics Data* 9, 40–41. doi:10.1016/j.gdata.2016.04.001.

Murugesan, P., Aswathy, G. M., Kumar, K. S., Masilamani, P., Kumar, V., and Ravi, V. (2017). Oil palm (Elaeis guineensis) genetic resources for abiotic stress tolerance: A review. *Indian J. Agric. Sci.* 87, 571–579.

Mutwakil, M. Z., Hajrah, N. H., Atef, A., Edris, S., Sabir, M. J., Al-Ghamdi, A. K., et al. (2017). Transcriptomic and metabolic responses of Calotropis procera to salt and drought stress. *BMC Plant Biol.* 17. doi:10.1186/s12870-017-1155-7.

Nansamba, M., Sibiya, J., Tumuhimbise, R., Karamura, D., Kubiriba, J., and Karamura, E. (2020). Breeding banana (Musa spp.) for drought tolerance: A review. *Plant Breed.* 139, 685–696. doi:10.1111/pbr.12812.

Newton, R. J., and Goodin, J. R. (1989). “Moisture Stress Adaptation in Shrubs,” in *The Biology and Utilization of Shrubs*, ed. C. McKell (Academic Press), 365–383. doi:https://doi.org/10.1016/B978-0-12-484810-8.50024-5.

Ntoulas, N., Nektarios, P. A., Spaneas, K., and Kadoglou, N. (2012). Semi-extensive green roof substrate type and depth effects on Zoysia matrella “Zeon” growth and drought tolerance under different irrigation regimes. *Acta Agric. Scand. Sect. B Soil Plant Sci.* 62, 165–173. doi:10.1080/09064710.2012.681391.

Okogbenin, E., Setter, T. L., Ferguson, M., Mutegi, R., Ceballos, H., Olasanmi, B., et al. (2013). Phenotypic approaches to drought in cassava: review. *Front. Physiol.* 4. doi:10.3389/fphys.2013.00093.

Patton, A. J., Schwartz, B. M., and Kenworthy, K. E. (2017). Zoysiagrass (Zoysia spp.) history, utilization, and improvement in the United States: A review. *Crop Sci.* 57. doi:10.2135/cropsci2017.02.0074.

Paudel, I., Gerbi, H., Zisovich, A., Sapir, G., Ben-Dor, S., Brumfeld, V., et al. (2019). Drought tolerance mechanisms and aquaporin expression of wild vs. cultivated pear tree species in the field. *Environ. Exp. Bot.* 167. doi:10.1016/j.envexpbot.2019.103832.

Pidgeon, J. D., Ober, E. S., Qi, A., Clark, C. J. A., Royal, A., and Jaggard, K. W. (2006). Using multi-environment sugar beet variety trials to screen for drought tolerance. *F. Crop. Res.* 95, 268–279. doi:10.1016/j.fcr.2005.04.010.

Plants for a Future, A. (2020). Hevea brasiliensis - (A.Juss.) Meull. Available at: https://pfaf.org/user/Plant.aspx?LatinName=Hevea+brasiliensis.

Safronov, O., Kreuzwieser, J., Haberer, G., Alyousif, M. S., Schulze, W., Al-Harbi, N., et al. (2017). Detecting early signs of heat and drought stress in Phoenix dactylifera (date palm). *PLoS One* 12. doi:10.1371/journal.pone.0177883.

Sahitya, U. L., Krishna, M. S. R., and Suneetha, P. (2019). Integrated approaches to study the drought tolerance mechanism in hot pepper (Capsicum annuum L.). *Physiol. Mol. Biol. Plants* 25, 637–647. doi:10.1007/s12298-019-00655-7.

Sapeta, H., Lourenço, T., Lorenz, S., Grumaz, C., Kirstahler, P., Barros, P. M., et al. (2016). Transcriptomics and physiological analyses reveal co-ordinated alteration of metabolic pathways in Jatropha curcas drought tolerance. *J. Exp. Bot.* 67, 845–860. doi:10.1093/jxb/erv499.

Schwab, K. B., and Heber, U. (1984). Thylakoid membrane stability in drought-tolerant and drought-sensitive plants. *Planta* 161, 37–45. doi:10.1007/BF00951458.

Shan-An, H., Gu, Y., and Zi-Jie, P. (1997). “Resources and Prospects of Ginkgo biloba in China,” in *Ginkgo Biloba A Global Treasure* (Springer Japan), 373–383. doi:10.1007/978-4-431-68416-9_28.

Sofo, A. (2011). Drought stress tolerance and photoprotection in two varieties of olive tree. *Acta Agric. Scand. Sect. B Soil Plant Sci.* 61, 711–720. doi:10.1080/09064710.2010.545071.

Su, X., Wei, F., Huo, Y., and Xia, Z. (2017). Comparative Physiological and Molecular Analyses of Two Contrasting Flue-Cured Tobacco Genotypes under Progressive Drought Stress. *Front. Plant Sci.* 8. doi:10.3389/fpls.2017.00827.

Sunil, M., Hariharan, A. K., Nayak, S., Gupta, S., Nambisan, S. R., Gupta, R. P., et al. (2014). The draft genome and transcriptome of amaranthus hypochondriacus: A C4 dicot producing high-lysine edible pseudo-cereal. *DNA Res.* 21, 585–602. doi:10.1093/dnares/dsu021.

Tang, S., Dong, Y., Liang, D., Zhang, Z., Ye, C. Y., Shuai, P., et al. (2015). Analysis of the Drought Stress-Responsive Transcriptome of Black Cottonwood (Populus trichocarpa) Using Deep RNA Sequencing. *Plant Mol. Biol. Report.* 33, 424–438. doi:10.1007/s11105-014-0759-4.

Tezara, W., Colombo, R., Coronel, I., and Marín, O. (2011). Water relations and photosynthetic capacity of two species of Calotropis in a tropical semi-arid ecosystem. *Ann. Bot.* 107, 397–405. doi:10.1093/aob/mcq245.

Vanburen, R., Bryant, D., Edger, P. P., Tang, H., Burgess, D., Challabathula, D., et al. (2015). Single-molecule sequencing of the desiccation-tolerant grass Oropetium thomaeum. *Nature* 527, 508–511. doi:10.1038/nature15714.

Varshney, R. K., Chen, W., Li, Y., Bharti, A. K., Saxena, R. K., Schlueter, J. A., et al. (2012). Draft genome sequence of pigeonpea (Cajanus cajan), an orphan legume crop of resource-poor farmers. *Nat. Biotechnol.* 30, 83–89. doi:10.1038/nbt.2022.

Varshney, R. K., Thudi, M., Nayak, S. N., Gaur, P. M., Kashiwagi, J., Krishnamurthy, L., et al. (2014). Genetic dissection of drought tolerance in chickpea (Cicer arietinum L.). *Theor. Appl. Genet.* 127, 445–462. doi:10.1007/s00122-013-2230-6.

Vaughan, D. A., Morishima, H., and Kadowaki, K. (2003). Diversity in the Oryza genus. *Curr. Opin. Plant Biol.* 6, 139–146. doi:10.1016/S1369-5266(03)00009-8.

Wan Nazri, W. B., Ezdiani, Z. N., Romainor, M. M., Erma, K. S., Jurina, J., and Noor Fadzlina, I. Z. A. (2014). Effect of fibre loading on mechanical properties of durian skin fibre composite. *J. Trop. Agric. Food Sci.* 42, 169–174.

Wan, T., Liu, Z.-M., Li, L.-F., Leitch, A. R., Leitch, I. J., Lohaus, R., et al. (2018a). A genome for gnetophytes and early evolution of seed plants. *Nat. Plants* 4, 82–89. doi:10.1038/s41477-017-0097-2.

Wan, X. L., Zhou, Q., Wang, Y. Y., Wang, W. E., Bao, M. Z., and Zhang, J. W. (2015). Identification of heat-responsive genes in carnation (Dianthus caryophyllus L.) by RNA-seq. *Front. Plant Sci.* 6. doi:10.3389/fpls.2015.00519.

Wan, X., Zou, L. H., Zheng, B. Q., Tian, Y. Q., and Wang, Y. (2018b). Transcriptomic profiling for prolonged drought in dendrobium catenatum. *Sci. Data* 5, 1–9. doi:10.1038/sdata.2018.233.

Wang, C. J., Yang, W., Wang, C., Gu, C., Niu, D. D., Liu, H. X., et al. (2012). Induction of Drought Tolerance in Cucumber Plants by a Consortium of Three Plant Growth-Promoting Rhizobacterium Strains. *PLoS One* 7. doi:10.1371/journal.pone.0052565.

Wang, N., Zhang, W., Qin, M., Li, S., Qiao, M., Liu, Z., et al. (2017). Drought tolerance conferred in soybean (Glycine max. L) by GmMYB84, a novel R2R3-MYB transcription factor. *Plant Cell Physiol.* 58, 1764–1776. doi:10.1093/pcp/pcx111.

Wei, H., Feng, F., Lou, Q., Xia, H., Ma, X., Liu, Y., et al. (2016). Genetic determination of the enhanced drought resistance of rice maintainer HuHan2B by pedigree breeding. *Sci. Rep.* 6, 1–11. doi:10.1038/srep37302.

Whitmore, J. S. (2000). “Vegetables for Drought-Prone Areas,” in *Drought Management on Farmland* (Springer, Dordrecht), 106–111. doi:10.1007/978-94-015-9562-9_10.

Wilts, B. D., Rudall, P. J., Moyroud, E., Gregory, T., Ogawa, Y., Vignolini, S., et al. (2018). Ultrastructure and optics of the prism-like petal epidermal cells of *Eschscholzia californica* (California poppy). *New Phytol.* 219, 1124–1133. doi:10.1111/nph.15229.

Wiriya-Alongkorn, W., Spreer, W., Ongprasert, S., Spohrer, K., Pankasemsuk, T., and Müller, J. (2013). Detecting drought stress in longan tree using thermal imaging. *Maejo Int. J. Sci. Technol.* 7, 166–180.

Wu, W., Ng, W. L., Yang, J. X., Li, W. M., and Ge, X. J. (2018a). High cryptic species diversity is revealed by genome-wide polymorphisms in a wild relative of banana, Musa itinerans, and implications for its conservation in subtropical China. *BMC Plant Biol.* 18, 1–11. doi:10.1186/s12870-018-1410-6.

Wu, Z. Z., Ying, Y. Q., Zhang, Y. Bin, Bi, Y. F., Wang, A. K., and Du, X. H. (2018b). Alleviation of drought stress in Phyllostachys edulis by N and P application. *Sci. Rep.* 8.

Xiao, L., Yang, G., Zhang, L., Yang, X., Zhao, S., Ji, Z., et al. (2015). The resurrection genome of Boea hygrometrica: A blueprint for survival of dehydration. *Proc. Natl. Acad. Sci. U. S. A.* 112, 5833–5837. doi:10.1073/pnas.1505811112.

Yang, R., Jarvis, D. E., Chen, H., Beilstein, M. A., Grimwood, J., Jenkins, J., et al. (2013). The Reference Genome of the Halophytic Plant Eutrema salsugineum. *Front. Plant Sci.* 4. doi:10.3389/fpls.2013.00046.

Yang, W., Wang, K., Zhang, J., Ma, J., Liu, J., and Ma, T. (2017a). The draft genome sequence of a desert tree Populus pruinosa. *Gigascience* 6. doi:10.1093/gigascience/gix075.

Yang, X., Hu, R., Yin, H., Jenkins, J., Shu, S., Tang, H., et al. (2017b). The Kalanchoë genome provides insights into convergent evolution and building blocks of crassulacean acid metabolism. *Nat. Commun.* 8. doi:10.1038/s41467-017-01491-7.

Yang, Z., Dai, Z., Lu, R., Wu, B., Tang, Q., Xu, Y., et al. (2017c). Transcriptome Analysis of Two Species of Jute in Response to Polyethylene Glycol (PEG)- induced Drought Stress. *Sci. Rep.* 7.

Yasar, F., Uzal, O., Kose, S., Yasar, O., and Ellialtioglu, S. (2014). Enzyme activities of certain pumpkin (Cucurbita spp) species under drought stress. *Fresenius Environ. Bull.* 23, 1093–1099.

Zhang, G. Q., Liu, K. W., Li, Z., Lohaus, R., Hsiao, Y. Y., Niu, S. C., et al. (2017). The Apostasia genome and the evolution of orchids. *Nature* 549, 379–383. doi:10.1038/nature23897.

Zhang, H., Gong, G., Guo, S., Ren, Y., Xu, Y., and Ling, K.-S. (2011). Screening the USDA Watermelon Germplasm Collection for Drought Tolerance at the Seedling Stage. *HortScience* 46, 1245–1248.

Zhang, L., Wu, M., Teng, Y., Jia, S., Yu, D., Wei, T., et al. (2019). Overexpression of the Glutathione Peroxidase 5 (RcGPX5) Gene From Rhodiola crenulata Increases Drought Tolerance in Salvia miltiorrhiza. *Front. Plant Sci.* 9. doi:10.3389/fpls.2018.01950.
